# Severe acute respiratory syndrome coronavirus 2 pathology and cell tropism in tongue tissues of COVID-19 autopsies

**DOI:** 10.3389/fcimb.2024.1394721

**Published:** 2024-06-21

**Authors:** Longda Ma, Qian Liu, Manli Wang, Liang Liu, Zhihong Hu, Yiwu Zhou, Jia Liu

**Affiliations:** ^1^ Department of Forensic Medicine, Tongji Medical College of Huazhong University of Science and Technology, Wuhan, China; ^2^ State Key Laboratory of Virology, Wuhan Institute of Virology, Center for Biosafety Mega-Science, Chinese Academy of Sciences, Wuhan, China

**Keywords:** severe acute respiratory syndrome coronavirus 2, taste dysfunction, autopsied tongue, pathogenesis, cell tropism, receptor distribution

## Abstract

Since 2019, Coronavirus Disease 2019(COVID-19) has affected millions of people worldwide. Except for acute respiratory distress syndrome, dysgeusis is also a common symptom of COVID-19 that burdens patients for weeks or permanently. However, the mechanisms underlying taste dysfunctions remain unclear. Here, we performed complete autopsies of five patients who died of COVID-19. Integrated tongue samples, including numerous taste buds, salivary glands, vessels, and nerves were collected to map the pathology, distribution, cell tropism, and receptor distribution of severe acute respiratory syndrome coronavirus 2 (SARS-CoV-2) in the tongue. Our results revealed that all patients had moderate lymphocyte infiltration around the salivary glands and in the lamina propria adjacent to the mucosa, and pyknosis in the epithelia of taste buds and salivary glands. This may be because the serous acini, salivary gland ducts, and taste buds are the primary sites of SARS-CoV-2 infection. Multicolor immunofluorescence showed that SARS-CoV-2 readily infects Keratin (KRT)^7+^ taste receptor cells in taste buds, secretory cells in serous acini, and inner epithelial cells in the ducts. The major receptors, angiotensin-converting enzyme 2 (ACE2) and transmembrane protease serine subtype 2 (TMPRSS2), were both abundantly expressed in these cells. Viral antigens and receptor were both rarely detected in vessels and nerves. This indicates that SARS-CoV-2 infection triggers pathological injury in the tongue, and that dysgeusis may be directly related to viral infection and cellular damage.

## 1 Introduction

The worldwide pandemic of Coronavirus Disease 2019 (COVID-19) caused by severe acute respiratory syndrome coronavirus 2 (SARS-CoV-2) poses a serious public health threat worldwide. COVID-19 mainly manifests as acute respiratory distress syndrome (Huang et al., 2020), but accumulating evidence has shown that oral symptoms have a relatively high incidence. A research involving 79 studies with data from 13,252 patients showed that taste disorders (48%), dry mouth (35%), and oral ulceration (21%) have been identified as the prevalent symptoms of COVID-19 (Tan et al., 2022; Gupta et al., 2023). Although COVID-19-related dysgeusia is usually transient, approximately 5% of patients develop taste disorders that persist for more than four weeks. So it has also been listed as a common symptom of “long COVID” (Srinivasan, 2021). In addition to affecting appetite, dysgeusis also affects nutrition and has other health consequences (Ferrulli et al., 2022).

Owing to its common oral manifestations, SARS-CoV-2 infection of the oral cavity has also attracted considerable attention. First, the presence of viral RNA in the saliva and gingiva was identified (Xu et al., 2020a; Gupta et al., 2021; Brahim Belhaouari et al., 2022). Numerous studies based on publicly available databases or normal human specimens have shown that the major receptors for SARS-CoV-2 entry, angiotensin-converting enzyme 2 (ACE2) and transmembrane protease serine subtype 2 (TMPRSS2), are widely expressed in the oral mucosa and salivary glands, indicating that oral tissues are susceptible to SARS-CoV-2 (Sakaguchi et al., 2020; Zhong et al., 2020; Xu et al., 2020a; Okada et al., 2021; Sawa et al., 2021; Drozdzik and Drozdzik, 2022; Henin et al., 2022). Using autopsied or postmortem biopsied salivary glands from deceased patients who had COVID-19, SARS-CoV-2 infection and local inflammatory infiltration have been identified in the salivary glands (Huang et al., 2021; Matuck et al., 2021). Almost simultaneously, Doyle et al., using biopsied fungiform papillae of the tongue from a patient during the course of COVID-19, demonstrated that type II taste receptor cells in taste buds, rather than other epithelia in the mucosa, were infected by SARS-CoV-2, but no alteration was observed in the taste bud (Doyle et al., 2021). Due to the limitations of biopsy sampling, only one out of four fungiform papillae containing fewer taste buds was analyzed in this study. In later studies of autopsied mucosa of the tongue, ACE2 and viral proteins were found to be strongly co-expressed in basal cells and the superficial epithelia of the mucosa (Tamiya et al., 2023), and destruction of taste buds in circumvallate papillae was also observed (Henin et al., 2022). These studies indicate that the oral cavity is veritably infected by SARS-CoV-2, and COVID-related oral symptoms may be associated with the direct or indirect injury of the virus to oral tissues. However, most studies have elaborated on one or two aspects of receptor expression, viral infection, or histology in one local oral structure, such as salivary glands, taste buds, or mucosa. Systematic studies on pathology and viral infection based on COVID-19 autopsies are limited, especially in autopsied tongues. Therefore, the mechanisms underlying taste impairment remain unclear.

The tongue is the major organ in the oral cavity responsible for taste sensation. Taste is first discriminated by taste receptor cells within the taste buds. Half of taste buds are buried in the mucosal layer of circumvallate papillae then in the foliate and fungiform papillae of the tongue. Each taste bud is supplied by nerve fibers, capillary arteries, and veins. Taste buds receive taste signals and transmit them to the taste cortex via the V/VII/IX/X cranial nerve (Thomas et al., 2022). Under the mucous layer of circumvallate papillae, abundant salivary glands (including serous and mucous acini) are located in the lamina propria and the muscular layer. Salivary glands secrete saliva to protect the mucosa and keep the tongue moist and clean, so that taste buds can respond quickly to the stimulation of tastes (Zhang et al., 2016).

To provide a more comprehensive understanding of SARS-CoV-2 pathology and infection of the tongue, we collected tongue specimens from five individuals who had died due to COVID-19 by complete autopsy. The specimens contained abundant taste buds of circumvallate papillae in the mucosa layer, and blood vessels, nerves, and salivary glands in the lamina propria and muscular layer. We studied SARS-CoV-2 infection and pathology in various tongue structures, and detected SARS-CoV-2 cell tropism and receptor distribution *in situ* using multicolor immunofluorescence (IF).

## 2 Materials and methods

### 2.1 Patients and samples

Autopsy tongue samples were obtained from five deceased patients who had COVID-19 and who were confirmed to be SARS-CoV-2 positive by polymerase chain reaction (PCR) testing of nasopharyngeal swabs and chest computed tomography scans during the first COVID-19 pandemic wave in Wuhan. Written informed consent was obtained from the patient’s family before autopsy. Autopsies were conducted according to the regulations issued by the National Health Commission of China and approved by the ethics committee of Wuhan Jinyintan Hospital (permission number: KY-2020–15.01). Tongue tissues around the sulcus terminalis of the tongue, a shallow V-shaped groove that distinguishes the front 2/3 of the tongue from the back 1/3, was collected. The samples were fixed in 10% formalin and analyzed using hematoxylin and eosin, immunohistochemical (IHC), and IF staining. The control sample was obtained from four individuals who died acutely from accidents or sudden death. The control samples were confirmed to be SARS-CoV-2 negative by IHC using antibodies against SARS-CoV-2 receptor-binding domain of spike protein (anti-RBD, homemade) (Liu et al., 2020). The present study was performed on cadavers scheduled for autopsy, as required by the Chinese Law Institute, to define the cause of death. Written informed consent was obtained from the family members of the patient.

### 2.2 Hematoxylin and eosin staining

The samples were taken at the sulcus terminalis, with a size of approximately 2 cm (length, parallel to the sulcus terminalis)×0.5 cm(width)×1.5 cm(height). The samples were dehydrated, permeated, embedded in paraffin, and then cut into 2-μm thick sections with a paraffin microtome. After deparaffinization, the slices were stained with hematoxylin and eosin.

### 2.3 IHC staining

After deparaffinization, formalin-fixed paraffin-embedded sections were subjected to heat-mediated antigen retrieval in citrate buffer (pH 6.0) (Sigma-Aldrich) using a microwave. After cooling to 25°C, the endogenous peroxidase was quenched with 3% hydrogen peroxide. Then the sections were blocked by phosphate-buffered saline containing 5% bovine serum albumin at 25°C for 1 h, and the primary antibodies anti-RBD, CD3 (Abcam, ab5690), CD19 (Ser, GB11061), and CD68 (CST, 76437) were incubated for overnight at 4°C, respectively. After washing, the sections were incubated with horseradish peroxidase-conjugated goat anti-rabbit/mouse IgG antibody (Proteintech) at 25°C for 1 h, then stained using DAB staining kit (Servicebio, G1212) and counterstaining with hematoxylin. Images were captured using a digital camera under a light microscope (Thermo Fisher Scientific).

### 2.4 IF staining

After deparaffinization, slices were blocked with 0.1% Sudan Black B (Sigma-Aldrich) for 15 min to reduce autofluorescence. The slices were repaired in citrate buffer by microwave irradiation for 30 min. After cooling to 25°C, the slices were blocked with phosphate-buffered saline containing 5% bovine serum albumin for 1 h at 25°C, and the primary antibody, including anti-RBD antibody (homemade), KRT5 (Abcam, ab64081), KRT7 (CST, 4465), ACE2 (Abcam, ab15348), and TMPRSS2 (Abcam, ab92323), were added and incubated overnight at 4°C. Then the washed sections were incubated with the secondary Alexa Fluor 488/555-conjugated goat anti-rabbit or mouse IgG (Abcam) for 2 h at 25°C. Images were captured using a digital camera under a light microscope (Thermo Fisher Scientific).

### 2.5 Multiplex IF assay

Multiplex fluorescence staining was performed using the Opal 7-color Manual IHC kit (Perkinelmer, NEL811001kt). After deparaffinization, antigen retrieval was performed by microwave irradiation in retrieval buffer according to the manufacturer’s instructions. The slices were then incubated with blocking buffer for 10 min at room temperature. Primary antibody was incubated for 1 h at 25°C, followed by staining using the horseradish peroxidase-conjugated secondary antibody and TSA-dendron-fluorophores. Primary and secondary antibodies were thoroughly removed by heating the sections in a retrieval buffer using microwaves. In serial operations, each antigen, including SARS-CoV-2 RBD, KRT5, KRT7, ACE2, and TMRPSS2, was labeled with distinct fluorophores, and the sections were imaged using a PerkinElmer VECTRA3 automatic quantitative analysis system.

## 3 Results

### 3.1 SARS-CoV-2 infection induced tongue damage

Tongue specimens were collected via complete autopsy from five deceased patients who had COVID-19. All patients were admitted to the hospital from January to February 2020 (first COVID-19 pandemic wave in Wuhan) and confirmed as SARS-CoV-2 positive by real time PCR (RT-PCR) of nasopharyngeal swabs and chest computed tomography scans (**Supplementary Table S1**). The study included two women and three men with a mean age of 60.8 years (range 51–72 years). The average survival time was 28.4 days (range 17–34 days). Autopsy was carried out in time, and the mean time lapse between death and autopsy was just 7.2 hours (range 4.5–9 hours) (**Supplementary Table S1**). The control patients died acutely (survival time <3 days) from accidents or sudden death with a mean age of 45 years (17–58 years), and the mean time lapse between death and autopsy was 180 hours (**Supplementary Table S1**).

**Figure 1:**
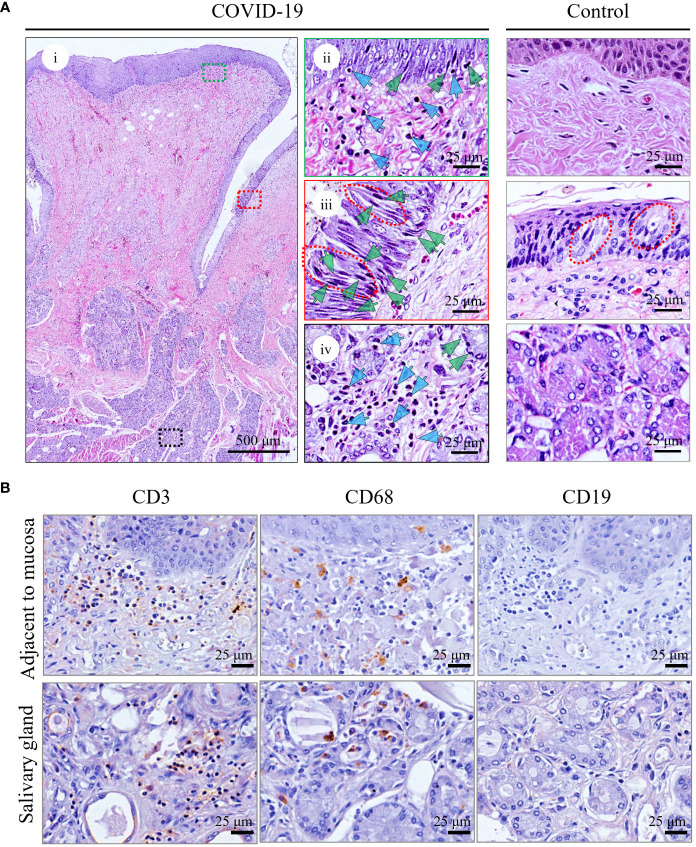
Histopathological analyses of tongue from representative death with COVID-19 (COVID-Case 3) or control (control 1). **(A)** Hematoxylin and Eosin staining of tongues. i showed a complete circumvallate papillae of the tongue and its deeply associated tissue from COVID-Case 3. The green and blue arrows indicate pyknosis and lymphocyte infiltration, respectively, in superficial part of the lamina propria (ii), taste buds (iii), and salivary glands (iv). The right panels (ii–iv) are enlarged images of the respective colored boxes on the left (i). Red lines in (iii) outline taste buds. **(B)** Immunohistochemical staining of inflammatory cells in the tongue of patients with COVID-19. CD3^+^ T lymphocytes, CD68^+^ macrophages, and CD19^+^ B lymphocytes were detected.

The overall pathological features of the tongue were analyzed in detail. Although no oral symptoms were recorded in the medical records, microscopic analyses revealed that all patients had moderate inflammatory cell infiltration (**Figure 1A**; **Supplementary Figure S1A**, blue arrows), particularly in the superficial part of the lamina propria adjacent to the mucosa (**Figure 1A**, ii; **Supplementary Figure S1A**, upper panel) and around the salivary glands (**Figure 1A**, iv; **Supplementary Figure S1A**, middle panel). In addition, necrosis or pyknosis (green arrows) in the epithelial cells of the mucosa (**Figure 1A**, ii and iii), taste buds (**Figure 1A**, iii; **Supplementary Figure S1A**, lower panel), and salivary glands (**Figure 1A**, iv; **Supplementary Figure S1A**, middle panel) were clearly observed. Mild edema with loose texture and slight staining in the superficial part of the lamina propria, especially adjacent to the taste buds (**Figure 1A**, iii; **Supplementary Figure S1A**, lower panel), was also detected. In the control tongues, the morphology of mucosa containing the taste buds and salivary glands is basically normal, and only mild inflammatory cell infiltration were observed (**Figure 1A**; **Supplementary Figure S1**).

To characterize the inflammatory responses to the presence of viral infection in the tongue, IHC staining of the infiltrating inflammatory cells was performed, and as shown in **Figure 1B**, varying amounts of CD3^+^ T lymphocytes and CD68^+^ macrophages aggregated in the superficial part of the lamina propria adjacent to mucosa and around the salivary glands, while CD19^+^ B lymphocytes were rarely detected. A very few inflammatory cells were detected in the controls (**Supplementary Figure S2**).

### 3.2 Taste buds and serous salivary glands were the major target of SARS-CoV-2

**Figure 2:**
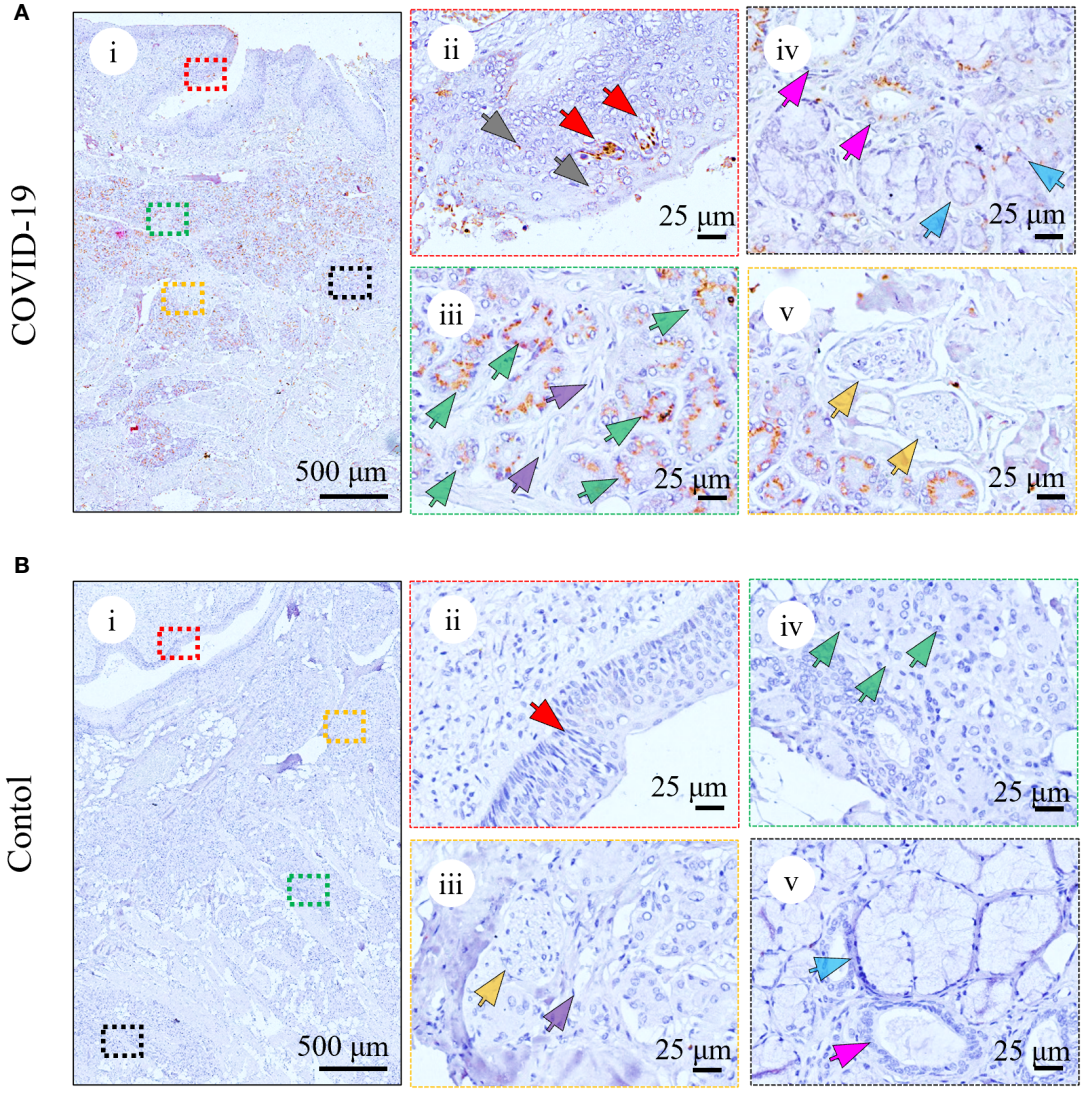
Detection of SARS-CoV-2 spike protein in the representative tongue from death with COVID-19 (COVID-Case 3) **(A)** or without COVID-19 (control 1) **(B)**. **(A)** Immunohistochemical analyses of SARS-CoV-2 infection in the patient with COVID-19 using anti-RBD antibody in taste buds (ii, red arrows), other epithelia of the mucosa (ii, grey arrows), serous acini (iii, green arrows), blood vessels (iii, purple arrows), salivary gland ducts (iv, magenta arrows), mucous acini (iv, blue arrows), and nerve fibers (v, yellow arrows). **(B)** Immunohistochemical analyses of SARS-CoV-2 infection in the control using anti-RBD antibody in taste buds (ii, red arrows), blood vessels (iii, purple arrows), nerve fibers (iii, yellow arrows), serous acini (iv, green arrows), salivary gland ducts (v, magenta arrows), and mucous acini (v, blue arrows). The right panels (ii–v) are enlarged images of the respective colored boxes on the left (i).

To investigate whether damage in the tongue in the patient was caused by a direct viral attack, IHC (**Figures 2A**; **Supplementary Figure S3**) and IF (data not shown) analyses were performed using antibodies against the RBD of spike protein of SARS-CoV-2. As shown in **Figures 2A** and **Supplementary Figure S3**, positive spike protein signals were observed in all five patients with COVID-19, while no viral antigen was detected in the tongues of the controls (**Figure 2B**; **Supplementary Figure S3B**; **Supplementary Table S1**). In the patients with COVID-19, as shown in **Figure 2A**; **Supplementary Figure S3A**, the virus antigens were primarily located in the serous acini (**Figure 2A**, iii; **Supplementary Figure S3A**, green arrows), followed by the taste buds (**Figure 2A**, ii; **Supplementary Figure S3A**, red arrows) and salivary gland ducts (**Figure 2A**, iv; **Supplementary Figure S3A**, magenta arrows). Only small amounts of viral spike protein resided in other epithelia of the mucosa (**Figure 2A**, ii, gray arrows) as well as mucous acini (**Figure 2A**, iv, blue arrows). In contrast, the virus was rarely detected in the blood vessels (**Figure 2A**, iii; **Supplementary Figure S3A**, purple arrows) or nerve fibers (**Figure 2A**, v; **Supplementary Figure S3A**, yellow arrows). The infection of SARS-CoV-2 in human tongues is summarized in **Supplementary Table S2**.

### 3.3 Multi-tongue cell types were infected

**Figure 3:**
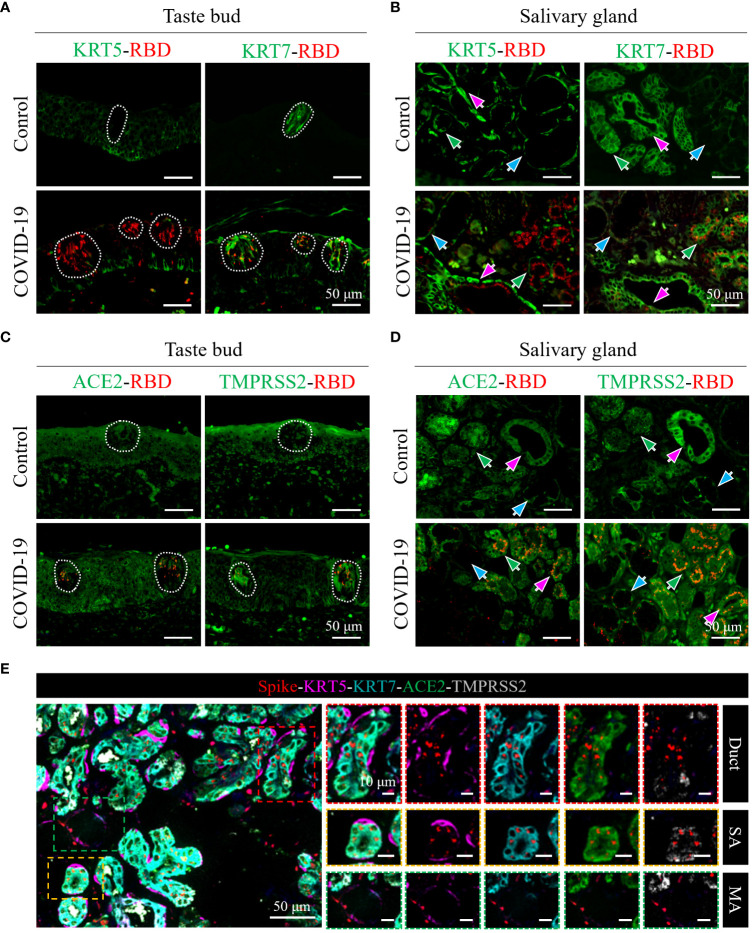
Characterization of cell tropism of SARS-CoV-2 and distribution of receptors. **(A, B)** Analysis of target cells of SARS-CoV-2 infection in the taste buds **(A)** and in the serous/mucous acini and salivary gland ducts **(B)**. White dotted lines indicate taste buds. KRT7 and KRT5 are cell markers of secretory luminal cells and ductal epithelial cells and outer myoepithelial/basal cells, respectively. **(C, D)** Colocalization analyses of SARS-CoV-2 spike protein (red) with ACE2 (green) or TMPRSS2 co-receptor (green) in the taste buds **(C)** and in the salivary glands **(D)**. **(E)** Multicolor immunofluorescence staining of SARS-CoV-2 spike protein (red), cell markers of secretory luminal cells and ductal epithelial cells (KRT7; cyan) and outer myoepithelial/basal cells (KRT5; magenta), ACE2 receptor (green), and TMPRSS2 co-receptor (gray). The right panels are enlarged images of the respective colored boxes on the left.

To ascertain the detailed SARS-CoV-2 cell tropism in tongue tissues, a colocalization analysis of viral spike proteins and individual cell markers was performed. In taste buds, which comprise inner taste receptor cells and outer stem cells (Iyer et al., 2021), KRT7^+^ taste receptor cells are the major target cells for SARS-CoV-2 infection, whereas KRT5^+^ stem cells are rarely infected (**Figure 3A**, lower panel). In the salivary glands, which are composed of KRT7^+^ inner secretory luminal cells and KRT5^+^ outer myoepithelial cells (Iyer et al., 2021), dual-colored IF (**Figure 3B**, lower panel, green arrows) revealed that SARS-CoV-2 primarily infected the KRT7^+^ secretory luminal cells of the serous acini, while viral antigens could not be detected in the KRT5^+^ outer myoepithelial cell layers. Similarly, in the salivary gland ducts, which contains the upper epithelia and lower basal cells (Iyer et al., 2021), it was also shown that KRT7^+^ epithelial cells of ducts, not the KRT5^+^ basal cells, were infected (**Figure 3B**, lower panel, magenta arrows). Additionally, a small number of KRT7^+^ mucous acini cells were infected with SARS-CoV-2 (**Figure 3B**, lower panel, blue arrows).

Furthermore, multicolored IF analysis confirmed that SARS-CoV-2 primarily targeted KRT7^+^ taste receptor cells, secretory luminal cells of the serous acini, and epithelial cells of the salivary gland ducts (**Figure 3E**).

### 3.4 ACE2^+^ */*TMPRSS2^+^-cells were mainly infected

ACE2 and TMPRSS2 receptors are major determinants for the cell tropism of SARS-CoV-2. To determine the relation between SARS-CoV-2 cell tropism and the distribution of receptors, we used dual-colored and multicolored IF analyses to determine the distribution of ACE2 and TMPRSS2 in relation to viral spike proteins in the tongue. As shown in **Figure 3C**, ACE2 and TMPRSS2 were both abundantly expressed in the taste receptor cells and other epithelia of the mucosa of the tongue; however, only taste receptor cells were primarily targeted by SARS-CoV-2. In taste buds, the expression of ACE2 and TMPRSS2 in COVID-19 cells was higher than that in the control group (**Figure 3C**).

In the serous acini and salivary gland ducts, ACE2 and TMPRSS2 were both abundantly expressed in KRT7^+^ secretory luminal cells and epithelial cells of duct, whereas they were sparingly expressed in KRT5^+^ outer myoepithelial cells and basal cells, respectively (**Figures 3D, E**, SA and duct). The expression levels of ACE2 and TMPRSS2 were both relatively lower in the mucous acini, especially that of ACE2 (**Figure 3D**). Consistently, the viral spike protein distribution corresponded to that of ACE2 and TMPRSS2 (**Figures 3D, E**).

In the vessels and nerves of the tongue of the control group, the expression of ACE2 and TMPRSS2 was relatively low (**Supplementary Figure S4**), and consistently rare viral antigens were detected in these structures (**Figure 2**). Collectively, these results potentially explain the high expression of the SARS-CoV-2 spike protein in taste buds, serous acini, and salivary gland ducts, and the low expression of the SARS-CoV-2 spike protein in mucous glands, vessels, and nerves in the tongue.

## 4 Discussion

**Figure 4:**
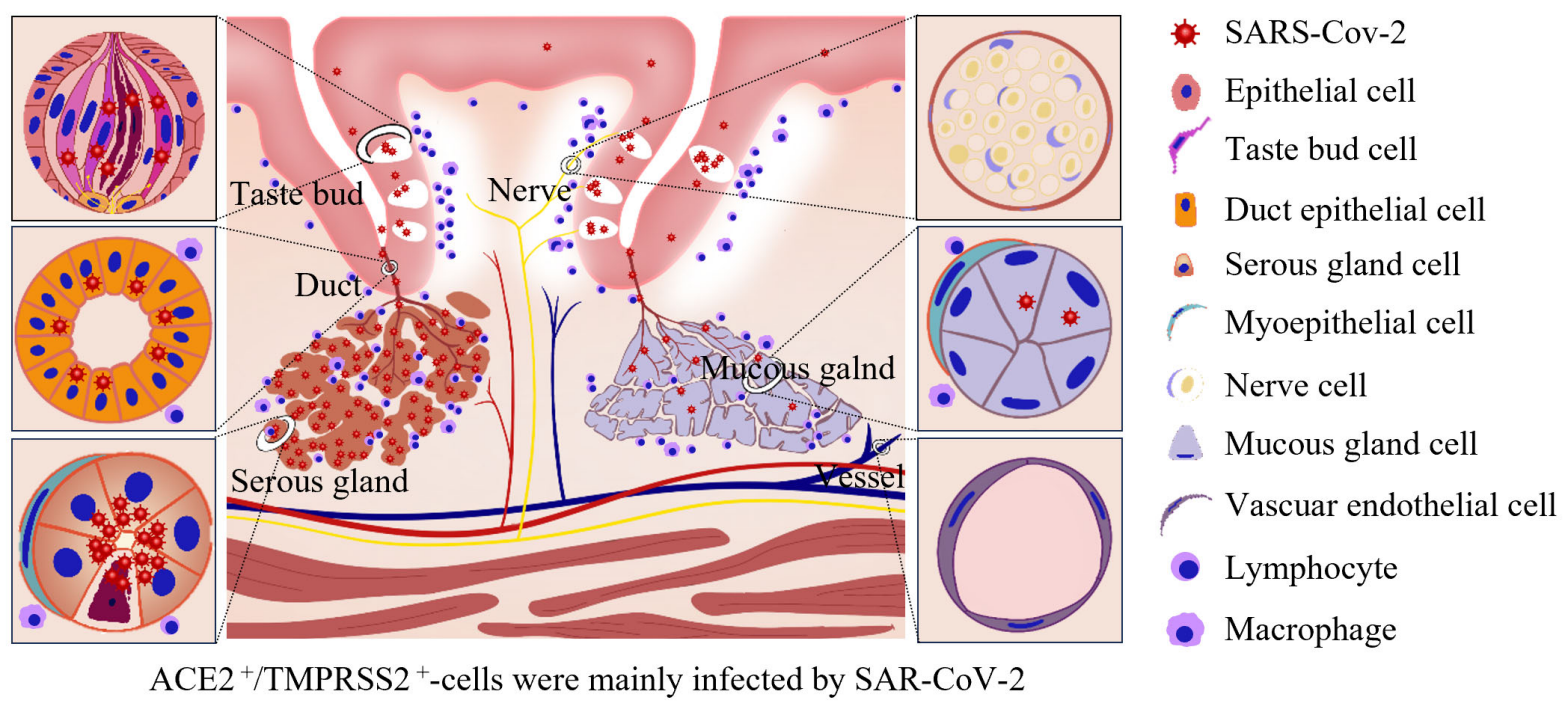
Schematic of pathology and SARS-CoV-2 infection in the human tongue. SARS-CoV-2 primarily infected the KRT7^+^ taste receptor cells in taste buds, secretory cells in serous acini, and salivary gland duct epithelial cells. ACE2 and TMPRSS2 were both abundantly expressed in these cells. The infection caused inflammatory cell infiltration adjacent to the mucosa and around the salivary glands, pyknosis in the epithelia of the taste buds and salivary glands and mild edema in the superficial part of the lamina propria, especially adjacent to the taste buds.

Dysgeusia is a common symptom of COVID-19 that burdens patients for weeks or even permanently in some cases and may be directly associated with cellular damage caused by virus infection (Amorim Dos Santos et al., 2021). However, the detailed mechanism underlying COVID-19 related dysgeusia remains unclear. Here, using the completely autopsied tongues of five individuals who died from COVID-19, we systematically revealed that SARS-CoV-2 primarily infected the taste buds in the mucosa as well as the serous acini and ducts of the salivary gland in the lamina propria and muscular layer of the tongue. The infection caused pathological damage to the tongue, mainly including inflammatory cell infiltration adjacent to the mucosa and around the salivary glands, as well as pyknosis in the epithelia of the taste buds and salivary glands. The virus primarily resides in the KRT7^+^ taste receptor cells in taste buds, secretory cells in serous acini, and salivary gland duct epithelial cells. ACE2 and TMPRSS2 were both abundantly expressed in these cells. A schematic diagram of the pathology and SARS-CoV-2 infection of the human tongue is summarized in **Figure 4**.

Among the oral symptoms, dysgeusia is the most common (Amorim Dos Santos et al., 2021). Our results showed that SARS-CoV-2 can directly infect taste receptor cells (**Figures 2**, **3**), cause structural disruption in the taste buds, and trigger obvious inflammatory infiltration and edema in the superficial part of the lamina propria under the taste buds (**Figure 1**). Taste buds are responsible for sensing and transmitting sweet, bitter, sour, and salty tastes. Infection and damage to taste receptor cells may impair taste recognition or conduction, resulting in weakened taste, or even loss (Srinivasan, 2021). Usually, dysgeusia resolves after the virus is cleared; however, it can persist in some patients and could last longer. This might be due to local inflammation around the taste buds, which not only induces apoptosis of taste receptor cells, but also disrupts the renewal cycle of taste bud stem cells (Wang et al., 2023).

Taste dysfunction is also a natural consequence of impaired tongue nerve function. A study revealed that SARS-CoV-2 can infect olfactory neurons *in vitro* and damage their chemosensory function (Lyoo et al., 2022). Additionally, SARS-CoV-2 RNA was also detected in the brain tissue of infected patients (Baig et al., 2020), suggesting that SARS-CoV-2 may have neurotropic properties. However, we did not find the SARS-CoV-2 spike protein or pathological injury in the nerve fibers of the tongue (**Figure 2A**). Consistent with this, the major viral receptors, ACE2 and TMPRSS2, were sparingly expressed in these nerve fibers (**Supplementary Figure S3**).

Approximately 32% and 35% of COVID-19 related oral symptoms are associated with salivary gland involvement and xerostomia, respectively (Gupta et al., 2023). We also found that salivary glands, especially serous acini, were susceptible to SARS-CoV-2 (**Figures 2**, **3**). Moderate lymphocyte infiltration was observed around the salivary glands (**Figure 1**; **Supplementary Figure S1**). This may affect saliva production and result in a dry mouth, which further influences tongue sensitivity to taste stimuli. In addition, saliva-containing viruses can be expelled from the duct into the oral cavity, which increases the risk of spreading the virus (Xu et al., 2020b; Gupta et al., 2021).

Studies have confirmed the expression of the major SARS-CoV-2 entry factors, ACE2 and TMPRSS2, in the oral mucosa, tongue, and salivary glands of healthy people; however, for a particular tissue or cell of the tongue, the results are contradictory (Sakaguchi et al., 2020; Huang et al., 2021; Sawa et al., 2021). For example, Sakaguchi et al. identified enriched expression of ACE2 and TMPRSS2 in taste buds, serous acini, and salivary gland ducts by IHC (Sakaguchi et al., 2020), whereas Sawa et al. detected ACE2 and TMPRSS2 in taste buds and TMPRSS2 in the salivary gland ducts by IFA (Sawa et al., 2021). The single-cell sequencing data of Huang et al. predicted the rare expression of ACE2 and TMPRSS2 in the salivary glands and ducts of healthy volunteers; however, they subsequently found that receptors and SARS-CoV-2 infection were more frequent in the salivary glands and ducts during COVID-19 autopsy using *in situ* hybridization (Huang et al., 2021). In this study, we analyzed ACE2 and TMPRSS2 expression in both non-COVID-19 and COVID-19 autopsies (**Figure 3**). Our results showed that in the non-COVID-19 and COVID-19 autopsy, ACE2 and TMPRSS2 were both abundantly expressed in taste buds, serous acini, and salivary gland ducts, where the viral antigens were also primarily located. In addition, ACE2 and TMPRSS2 were expressed at higher levels in the taste buds of patients with COVID-19 than in the control group (**Figure 3C**), indicating that SARS-CoV-2 infection might change the relative receptor expression. Furthermore, we used multicolor IF analysis to classify the expression of receptors and viral antigens in different tongue cells. ACE2 and TMPRSS2 were both abundantly expressed in KRT7^+^ taste receptor cells in taste buds, secretory cells in serous acini, and the salivary gland duct epithelia, consistent with the virus being mainly located in these cells.

Our investigation is circumscribed by several limitations. Foremost among these is the restricted number of autopsy specimens available for analysis. This limitation is mainly attributed to the exigencies encountered during the initial surge of the COVID-19 pandemic in Wuhan, characterized by constrained resources and a nascent comprehension of the disease’s pathophysiology. The acquisition of autopsy samples was notably challenging during this period. A secondary limitation stems is the absence of comprehensive clinical record about taste disorders. Early in the course of the COVID-19 outbreak, emphasis was predominantly placed on respiratory manifestations and severe life-threatening symptoms, relegating sensory alterations such as taste and olfactory dysfunctions to a secondary concern. So it renders it difficult to ascertain whether individuals within our study indeed experienced taste disorders. Nevertheless, our examination of postmortem specimens consistently revealed obvious pathological change indicative of SARS-CoV-2 infection. This indicates that our findings possess a degree of generalizability and suggests a potentially heightened prevalence of dysgeusia in the COVID-19 manifestations, surpassing current levels of recognition.

Overall, our study provides a comprehensive understanding of the pathology of SARS-CoV-2 infection in the tongue. The cell tropism of SARS-CoV-2 infection and relation between cell tropism and receptor distribution in human tongue was also clarified. These results indicate that SARS-CoV-2 infection may directly trigger pathological injury to the tongue and cause taste disorders.

## Data availability statement

The original contributions presented in the study are included in the article/**Supplementary Material**. Further inquiries can be directed to the corresponding authors.

## Ethics statement

The studies involving humans were approved by the ethics committee of Wuhan Jinyintan Hospital. The studies were conducted in accordance with the local legislation and institutional requirements. The participants provided their written informed consent to participate in this study.

## Author contributions

LM: Methodology, Investigation, Formal analysis, Data curation, Writing – original draft. QL: Project administration, Resources, Writing – review & editing. MW: Project administration, Conceptualization, Writing – review & editing. LL: Resources, Writing – review & editing. ZH: Writing – review & editing. YZ: Resources, Writing – review & editing. JL: Methodology, Formal analysis, Data curation, Conceptualization, Writing – review & editing, Writing – original draft.
