# Supplementary material for: Severe acute respiratory syndrome coronavirus 2 pathology and cell tropism in tongue tissues of COVID-19 autopsies
Source: Front Cell Infect Microbiol. 2024 Jun 21;14:1394721. doi: 10.3389/fcimb.2024.1394721 (PMC11224463; doi:10.3389/fcimb.2024.1394721)
Supplement: Supplementary file 1 [file DataSheet_1.docx]

**Severe Acute Respiratory Syndrome Coronavirus 2 Pathology and Cell Tropism in Tongue Tissues of Coronavirus Disease Autopsies**

Supplementary Appendix: Table of Contents

**Page**

[Supplementary Figures 2](#_Toc160191832)

[Figure S1 Histopathological analyses of tongue from representative death with (A) or without COVID-19 (B). 2](#_Toc160191833)

[Figure S2 Immunohistochemical staining of inflammatory cells in the tongue of representative patients without COVID-19. 3](#_Toc160191834)

[Figure S3 Detection of SARS-CoV-2 spike protein in the tongues from the other COVID-19 deaths (A) and control patients (B). 4](#_Toc160191835)

[Figure S4 The distribution of ACE2 (green) or TMPRSS2 (green) in the vessels and nerves from the non-COVID-19. The white dotted lines indicated blood vessels or nerves. 5](#_Toc160191836)

[Supplementary Tables 6](#_Toc160191837)

[Table S1. The information of all included patients with coronavirus (COVID-19). 6](#_Toc160191838)

[Table S2. Immunohistochemical analysis of severe acute respiratory syndrome coronavirus 2 spike protein in the tongue samples of individuals who died from coronavirus. 6](#_Toc160191839)

# Supplementary Figures


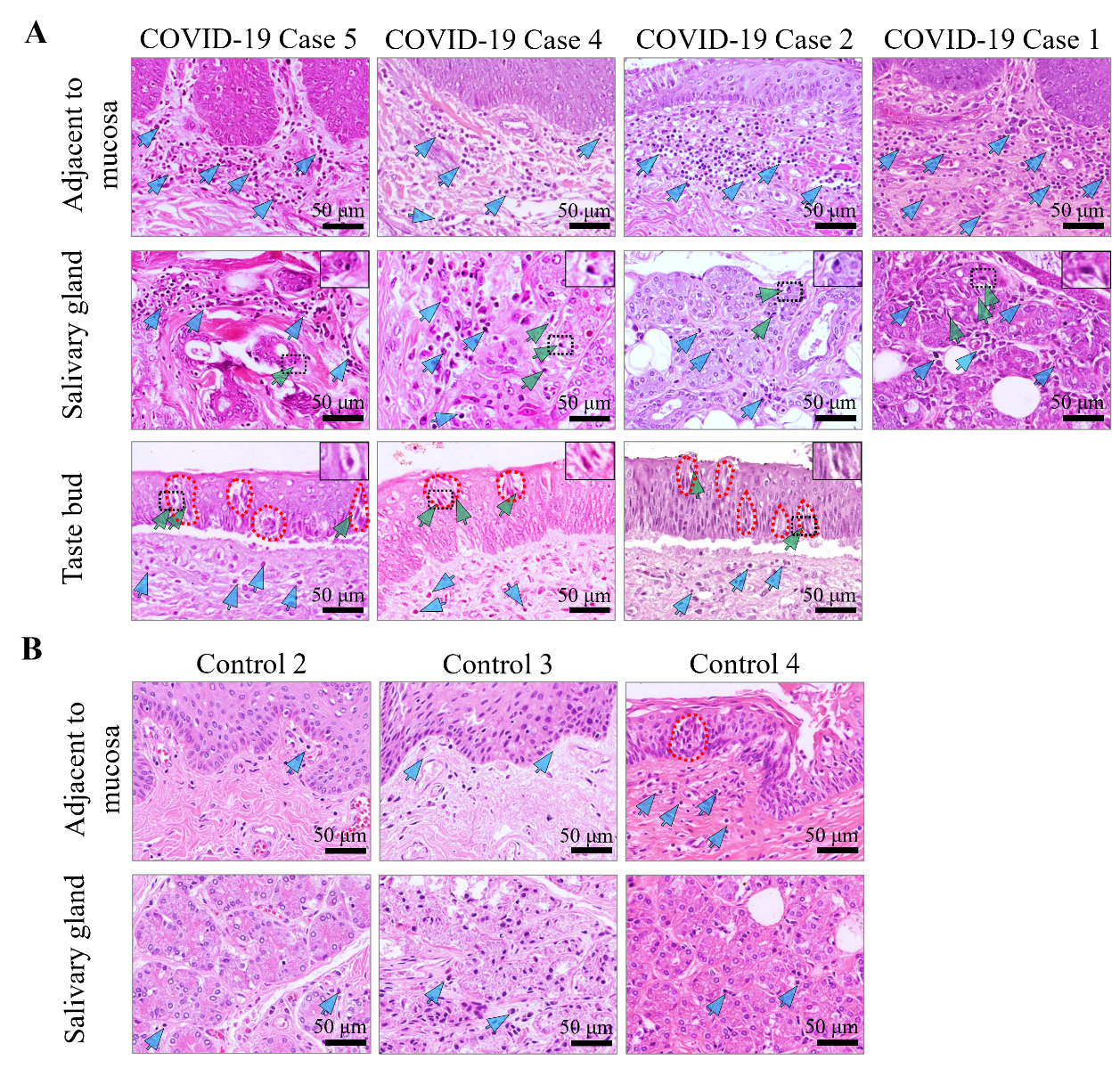


Figure S1 Histopathological analyses of tongue from representative death with (A) or without COVID-19 (B). Hematoxylin and eosin staining of tongues from the other COVID-19 deaths. The green, and blue arrows indicate pyknosis and lymphocyte infiltration, respectively, and red lines outline a taste bud. The inserted figures in the upper right corner, showing the pyknosis, are enlarged images of the black boxes on the left.


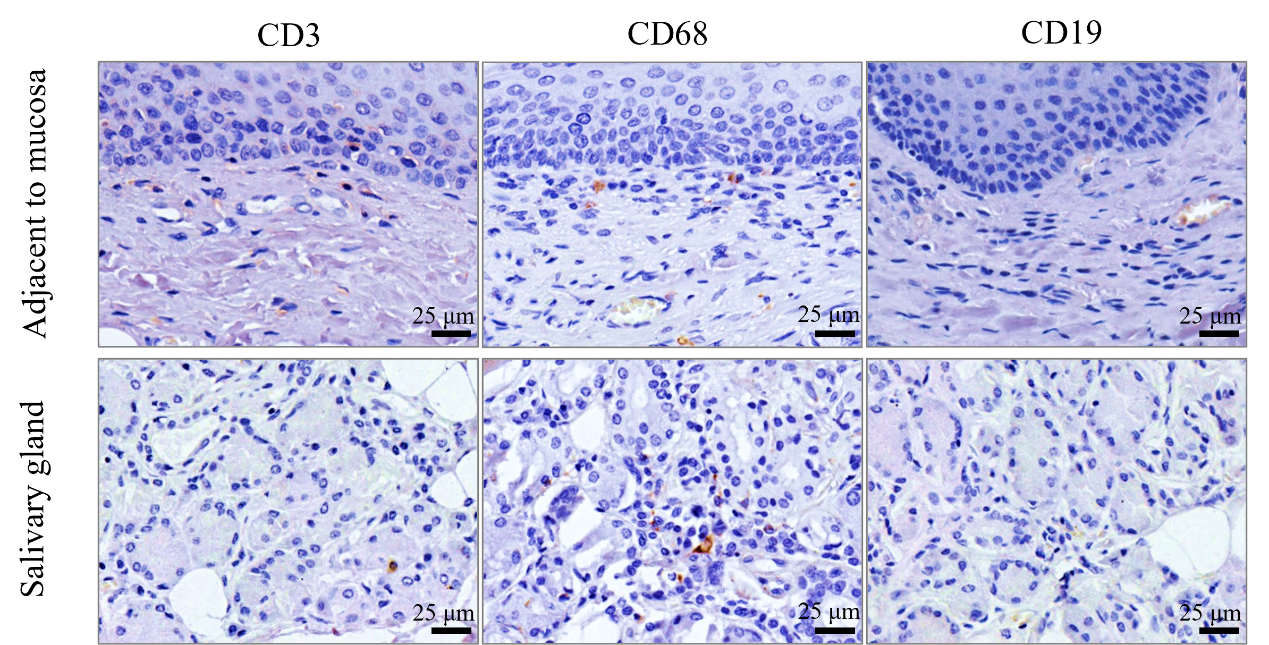


Figure S2 Immunohistochemical staining of inflammatory cells in the tongue of representative patients without COVID-19. CD3^+^ T lymphocytes, CD68^+^ macrophages, and CD19^+^ B lymphocytes were detected.


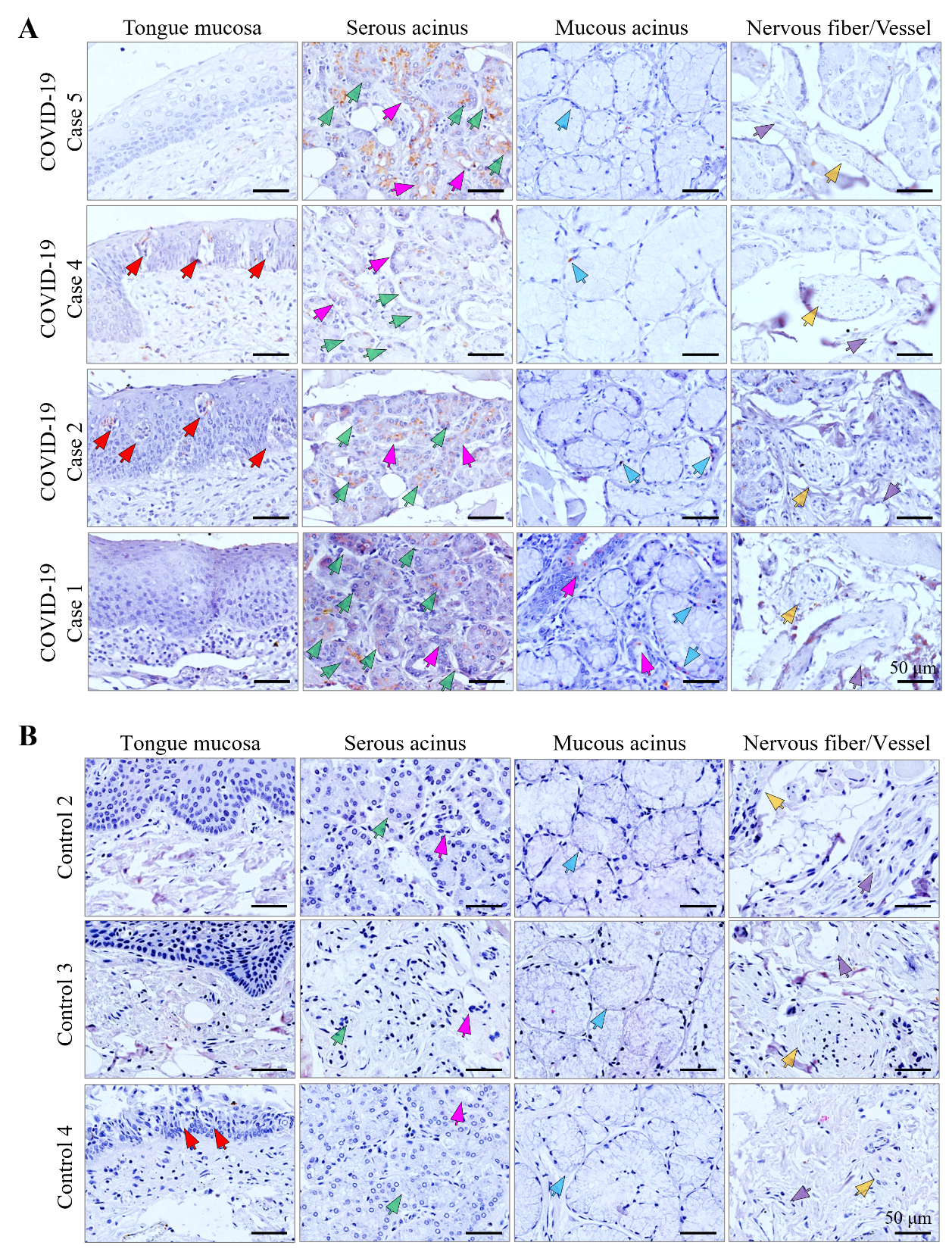


Figure S3 Detection of SARS-CoV-2 spike protein in the tongues from the other COVID-19 deaths (A) and control patients (B). Immunohistochemical analyses of SARS-CoV-2 infection in taste buds (red arrows), serous acini (green arrows), blood vessels (purple arrows), salivary gland ducts (magenta arrows), mucous acini (blue arrows), and nerve fibers (yellow arrows).


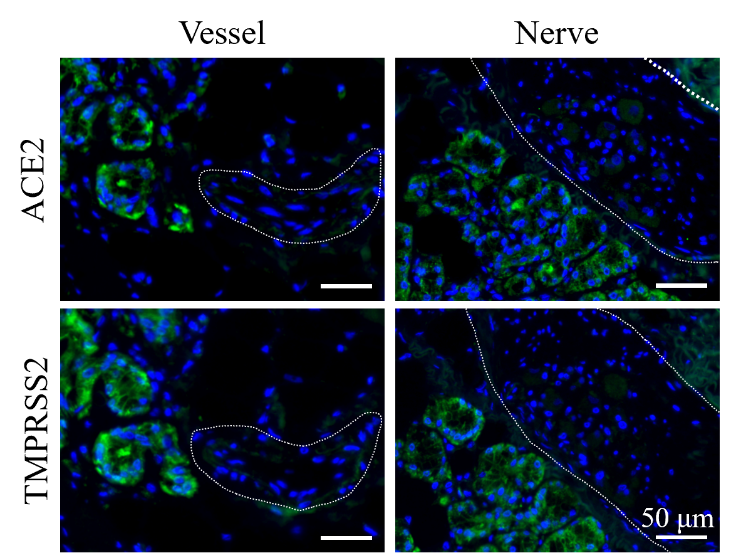


Figure S4 The distribution of ACE2 (green) or TMPRSS2 (green) in the vessels and nerves from the non-COVID-19. The white dotted lines indicated blood vessels or nerves.

Supplementary Tables

Table S1. The information of all included patients with coronavirus (COVID-19).

| Cases | Gender/Age | Admission time | Survival time (days) | Time lapse between autopsy and death (hours) | Viral RNA(RT-PCR) or protein (IHC) detection | Death causes |
| --- | --- | --- | --- | --- | --- | --- |

| COVID-19 |
| --- |

| 1 | F/66 | 2020-2 | 17 | 8 | + | COVID-19 |
| --- | --- | --- | --- | --- | --- | --- |
| 2 | M/72 | 2020-1 | 32 | 7 | + | COVID-19 |
| 3 | F/53 | 2020-1 | 34 | 7.5 | + | COVID-19 |
| 4 | M/62 | 2020-2 | 31 | 9 | + | COVID-19 |
| 5 | M/51 | 2020-2 | 28 | 4.5 | + | COVID-19 |

| Non-COVID-19 (control) |
| --- |

| 1 | M/58 | 2021-3 | <1 | 48 | - | Traffic accident |
| --- | --- | --- | --- | --- | --- | --- |
| 2 | M/17 | \ | <1 | 48 | - | Sudden death |
| 3 | M/74 | 2023-12 | 3 | 528 | - | Traffic accident |
| 4 | M/31 | 2021-3 | <1 | 96 | - | Alcohol poisoning and traffic accident |

Note: \ indicated the patient have not be hospitalized.

Table S2. Immunohistochemical analysis of severe acute respiratory syndrome coronavirus 2 spike protein in the tongue samples of individuals who died from coronavirus.

| Case | Mucosa | | Salivary gland | | | Vessel | Nerve |
| --- | --- | --- | --- | --- | --- | --- | --- |
|  | Taste bud | Other epithelium | Serous acinus | Mucous acinus | Duct |  |  |
| 1 | \ | - | ++ | + | ++ | - | - |
| 2 | +++ | - | ++ | + | ++ | - | - |
| 3 | ++ | + | +++ | + | +++ | - | - |
| 4 | +++ | - | + | - | + | - | - |
| 5 | \ | - | +++ | + | +++ | - | - |

Note: \ indicated no taste bud was observed. + indicates 1% to 25% corresponding tissues were infected; ++ indicates 26% to 50% corresponding tissues were infected; +++ indicates 51% to 75% corresponding tissues were infected.
